# The Social-Medical Network: Integration of Personal and Professional Healthcare Networks

**DOI:** 10.1093/geroni/igaf122.572

**Published:** 2025-12-31

**Authors:** Catherine Clair, Janiece Taylor, Karin Tobin

**Affiliations:** Johns Hopkins School of Nursing, Baltimore, Maryland, United States; Johns Hopkins University, Baltimore, Maryland, United States; Johns Hopkins Bloomberg School of Public Health, Baltimore, Maryland, United States

## Abstract

Older adults living with Type 2 diabetes may have two healthcare networks: Personal (family, friends, neighbors) and professional (healthcare providers). The social-medical network is the larger care network comprised these subnetworks and their integration. We aimed to 1) explore the characteristics of the subnetworks and social-medical network and 2) regress emergency department utilization on social-medical network integration. We conducted a cross-sectional observational study, which included a social network inventory, with community-dwelling older adults (65+ years) living with Type 2 diabetes. We ran a negative binomial regression of emergency department utilization on social-medical network integration, adjusting for the number of additional chronic conditions, having one personal alter, and an interaction term between the primary independent variable and having one personal alter. We completed data collection with 122 individuals. The analytic sample (n = 98) was 71.4% female (n = 70) and 58.2% non-White (n = 57). The mean age of participants was 73.79 ± 6.01 years. In adjusted analyses, for each 1-unit increase in integration, older adults with two or more personal alters had 0.43 times the incidence rate of emergency department visit (95% CI: 0.07-2.62) when controlling for other covariates of interest. The direction of the association was reversed for older adults with only one personal alter (IRR: 1.76, 95% CI: 0.36-8.55). Social-medical network integration had a protective effect on emergency department visit for older adults with two or more family and friends involved in their care. Integration of family and friends with healthcare providers is critical to support the health and well-being of this population.

